# Long-term dementia risk in metabolic dysfunction-associated steatotic liver disease: a population-based study

**DOI:** 10.1007/s11011-026-01796-x

**Published:** 2026-02-02

**Authors:** Andreas Bartholdy, Kristine Frøsig Moseholm, Pernille Yde Nielsen, Nicolai J. Wewer Albrechtsen, Lise Lotte Gluud, Majken Karoline Jensen

**Affiliations:** 1 Department of Public Health, University of Copenhagen, Copenhagen, Denmark; 2 Department of Applied Mathematics and Computer Science, Technical University of Denmark, Kongens Lyngby, 2800 Denmark; 3 Department of Clinical Biochemistry, Copenhagen University Hospital – Bispebjerg, Copenhagen, Denmark; 4 Department of Clinical Medicine, Faculty of Health and Medical Sciences, University of Copenhagen, Copenhagen, Denmark; 5 Gastro Unit, Copenhagen University Hospital Hvidovre, Hvidovre, 2650 Denmark

**Keywords:** MASLD, Dementia, Epidemiology, Registry-based

## Abstract

Metabolic dysfunction-associated steatotic liver disease (MASLD) has been implicated in cognitive decline through vascular and metabolic pathways, yet evidence linking it to dementia remains inconsistent. We aimed to determine this association by addressing implications of selection, confounding and reverse causation. We conducted a nationwide, registry-based matched cohort study including all individuals diagnosed with MASLD in Denmark between 2000 and 2020, each with 5 age- and sex-matched references without liver disease. Dementia diagnoses were ascertained through hospital-, prescription-, and death registries. We estimated hazard ratios (HR) using cause-specific Cox regression, adjusting sequentially for comorbidities and socioeconomic status. Sensitivity analyses included age restrictions, delayed entry to address reverse causations, and a negative control outcome (Chronic Obstructive Pulmonary Disease (COPD)) to assess residual confounding from misclassification of alcohol consumption. We included 8,398 individuals with MASLD of which 174 developed dementia during follow-up and 41,990 references of which 641 developed dementia during follow-up. In unadjusted analyses, a MASLD diagnosis was associated with an increased risk of dementia (HR 1.40; 95% CI 1.17 – 1.67). However, the association attenuated after adjustment for comorbidities and socioeconomic factors (HR 1.12; 95% CI 0.94–1.34). Delayed entry analyses suggested possible reverse causation as HRs tended to increase, suggesting underestimation. Using COPD as a negative control outcome showed no increased risk in MASLD patients. Although MASLD was not independently associated with increased dementia risk, our findings suggest possible reverse causation, theoretically reflecting that cognitive decline may cause weight loss, leading to underestimating the association.

**Supplementary Information:**

The online version contains supplementary material available at 10.1007/s11011-026-01796-x.

## Introduction

 Metabolic dysfunction-associated steatoticliver disease (MASLD) is the most common cause of chronic liver disease worldwide, and is associated with metabolic disease risk factors such as obesity, hypertension, and diabetes (Younossi et al. 2025; Miao et al. 2024). Once considered a primarily hepatic condition, MASLD is now recognized as a multisystem disease with implications for cardiovascular and metabolic health (Targher et al. 2024). While previously only treatable through lifestyle interventions, several pharmacological treatments have recently shown promise in clinical trials (Sanyal et al. 2024; Harrison et al. 2025, 2024).

Dementia represents a growing global health challenge, currently affecting over 55 million people worldwide – a number projected to rise in the coming decades ((Nichols et al. 2022). Due to the lack of disease-modifying treatments, there is substantial interest in identifying modifiable risk factors and upstream mechanisms for disease development (Litke et al. 2021).

Animal models of MASLD have found neuroinflammation, microglial activation, and impaired synaptic plasticity, particularly in the hippocampus and prefrontal cortex, suggesting a link with cognitive decline (Medina-Julio et al. 2024; Termite et al. 2025). Systemic inflammation, altered lipid metabolism, and insulin resistance have been implicated in blood-brain barrier disruption and cerebral small vessel disease (Van Dyken and Lacoste 2018; Zhou et al. 2020). Disruptions in the gut–liver–brain axis may also contribute to neurodegeneration via microbial metabolites and cytokine signaling (Termite et al. 2025). Human neuroimaging studies show changes in functional connectivity involving the amygdala and prefrontal cortex in MASLD patients with cognitive impairment (Jin et al. 2024; Shu et al. 2023).

Epidemiological findings on the association between MASLD and dementia have been inconsistent. Most prospective cohort studies report no clear link to dementia (Xiao et al. 2022; Huang et al. 2023; Wang et al. 2022), though some suggest a relationship with cognitive decline (Parikh et al. 2024). Prospective studies, however, have generally had short follow-up times in relation to dementia development, and may be susceptible to participation bias. Registry-based studies, which may better reflect real-world clinical populations and more readily offer longer follow-up times, have yielded mixed results. Many were unable to adjust for socioeconomic factors known to influence both MASLD and dementia, and while residual confounding is frequently acknowledged, few studies have directly evaluated its influence (Jeong et al. 2022; Labenz et al. 2021; Shang et al. 2022). Furthermore, these studies often include only individuals aged 65 and older at the time of MASLD diagnosis to enhance diagnostic accuracy for dementia. However, enrolling newly diagnosed MASLD patients after age 65 can potentially introduce selection bias, as metabolic diseases such as MASLD, on average, are diagnosed earlier (Lin et al. 2022). Focusing solely on incident MASLD as an exposure for dementia may also lead to reverse causation, as weight loss preceding a diagnosis of dementia could lower the likelihood of a MASLD diagnosis (Koutoukidis et al. 2021; Singh-Manoux et al. 2018).

The aim of this study was to determine the association between MASLD and dementia in a large population-based cohort. By leveraging comprehensive registry data and applying a series of sensitivity analyses, we sought to address key methodological limitations in the existing literature – namely, residual confounding, reverse causation, and selection bias. In doing so, we aimed to generate more robust and generalizable estimates of risk and to inform the potential role of MASLD as a modifiable target in dementia prevention.

## Materials and methods

### Study design and data sources

This is a registry-based population-wide matched cohort study, comparing individuals with incident MASLD to a reference population free from liver disease. Information from various registries is made possible by the Danish civil registration system (Pedersen 2011). This study uses data from the Danish National Patient Registry (DNPR) which contain administrative and diagnostic information on all contacts between patients and Danish hospitals, the Psychiatric Central Research Registry (PCRR) which similar data but on psychiatric inpatient and outpatient contacts (fused with DNPR in 1995), the population registry, the education registry, the income registry, the prescription registry, and the Danish National Health Services Registry which contains administrative information (but not diagnoses) of primary care physician contacts in Denmark (Lynge et al. 2011; Mors et al. 2011; Jensen and Rasmussen 2011; Baadsgaard and Quitzau 2011; Pottegård et al. 2017; Andersen et al. 2011). Diagnosis codes are coded as ICD codes (ICD-8 prior to 1995 and ICD-10 hereafter). Prescription substances are coded as Anatomical Therapeutic Chemical Classification System (ATC) codes.

### Study population

The cohort comprised all patients diagnosed with MASLD between January 1, 2000, and December 31, 2020, and of age ≥ 18 at the time of diagnosis. We first selected all individuals with a first-time registration of an ICD-10 diagnostic code for MASLD (K760* or DK758), who were living in Denmark in the study period, and who did not have missing information on birth year or sex. We then assessed individuals for any diagnosis code suggesting alcohol or other competing etiology for chronic liver disease (see supplementary material for codes). To allow time for full clinical assessment, each individual was assessed for codes pertaining to an exclusion diagnosis at any time prior to initial diagnosis until 180 days after. The index date for the individual was thus set to 180 days after the initial diagnosis. Lastly, we excluded patients meeting the definition for dementia before the index date. We then performed individual matching with replacement. Individuals who met the exclusion criteria, but were free from liver disease at the specified index date, were included as population references. We matched five references to each individual with MASLD. References were further matched on sex and birth year. References who later developed MASLD were censored from the reference population at that time and matched with their own five references.

### Covariates

Covariates included type 2 diabetes, hypertension, and dyslipidemia, stroke, heart disease (coronary heart disease, atrial fibrillation, or heart failure), depression, highest educational attainment, cohabitation, income, and health care utilization prior to index date.

Type 2 diabetes, hypertension, and dyslipidemia were defined based on prescription drug purchases because they are often treated by a primary care physician, who does not report to DNPR. Hypertension and dyslipidemia were defined as having purchased an antihypertensive or lipid-modifying drug at least twice within 180 days. Type 2 diabetes was defined as having purchased an antidiabetic drug at least twice within 180 days and having at least one purchase of a non-insulin antidiabetic drug in the same period. For all three conditions, 180 days after the initial purchase was set as the date of developing the given condition. Prevalent stroke, cardiovascular disease, and depression were determined based on any ICD-10 or ICD-8 registration of primary or secondary diagnosis prior to index date (supplementary). Highest educational attainment was defined in accordance with the international standard classification of education, and grouped into primary, secondary, and tertiary education (International 2011). For education, we considered missingness equivalent to no completed education beyond primary education. Income was based on equivalized disposable income in the year prior to the index date, divided into quintiles based on the whole population for the given year. In cases where information from that year was not available, information from the year prior to that was used. If that was also not available, income was classified as missing. Cohabitation was defined as living alone or living with a partner (married or otherwise) according to information in the Population Statistics Registry. Health care utilization was calculated based on primary care contacts. We defined it as the mean number of contacts in the four years preceding the index date was calculated and then further categorized into low (less than five annual contacts), medium (5–9 annual contacts), and high (10 or more annual contacts) utilization.

### Outcomes

The primary outcome was dementia, defined as the first registration of primary or secondary diagnosis of dementia in DNPR or PCRR or first registration of ATC code for dementia medication in the Prescription Registry or a dementia diagnosis registered as the underlying cause of death on the death certificate. A previous study has found high validity of dementia diagnoses in the registries in DNPR and PCRR (Phung et al. 2007). The secondary outcome of vascular dementia was defined as the first registration of a primary or secondary diagnosis of vascular dementia in DNPR or PCRR.

### Statistics

For baseline characteristics, we calculated frequency and percentages in groups. Cumulative incidence plots were created based on the Aalen-Johansen estimator with death from other causes as a competing event. To comply with Statistics Denmark’s rules for the discretization of individual-level data, the visualized plot data were first aggregated in groups of three, such that each step in the plot represents three consecutive events. Crude incidence rates were calculated as the number of events per 10000 person-years with confidence intervals based on the Poisson distribution. To assess differences between the two groups, we performed stratified cause-specific Cox regression, censoring at death from other events, emigration, and end of follow-up. The proportional hazards assumption was assessed by plotting scaled Schoenfeld residuals against time. Missingness in income and cohabitation of less than 5% was handled by dummy variable adjustment. We performed three models, one crude, one adjusting for confounding comorbidities, and one further including socioeconomic variables. Previous literature has found dementia diagnoses to be less reliable in individuals below 65 (Salem et al. 2013). However, the mean age of diagnosis of MASLD is significantly younger than that. To avoid selection by only including individuals diagnosed with MASLD later in life, we included all cases in the main analysis but performed a sensitivity analysis only including risk time from age 65 onwards, excluding individuals who developed dementia before this time.

To evaluate the potential influence of unmeasured confounding, we incorporated a negative control outcome analysis (Lipsitch et al. 2010). A valid negative control outcome should share confounding structures with the primary outcome but should not be causally affected by the exposure. COPD meets these criteria because it is strongly influenced by smoking and other lifestyle-related health factors that tend to correlate with alcohol use, allowing it to capture some of the confounding structure relevant for MASLD and dementia, while MASLD is not expected to have a causal effect on COPD. For the negative control outcome, we therefore used chronic obstructive pulmonary disease (COPD), defined as the first primary or secondary diagnosis in DNPR (see supplementary). In this context, a positive association between MASLD and COPD would suggest residual lifestyle-related confounding, whereas a null association would support the robustness of the primary findings. We repeated the main modeling strategy with COPD as the outcome.

We also estimated an adjusted hazard rate for all-cause mortality, which is registered in the cause of death registry, and performed sub-analyses for vascular dementia specifically. To address possible sex differences, we also performed the main analysis in strata of sex. To assess possible reverse causation, we introduced a lag time analysis with 5, 10, and 15 years of delayed entry. These analyses were conducted by removing all individuals with an event (dementia or competing event) or with censoring within the specified timeframe such that we were only analysing (conditioning on) events happening at least the specified amount of time after initial inclusion. For example, with 10 years delay we only included individuals who died, received their first dementia diagnosis or were censored at least 10 years after initial inclusion, effectively calculating only the hazard ratio (HR) from this point on. This removes early events where reverse causation is likely to be most pronounced. This analysis was repeated for COPD as the outcome. Finally, we did a sensitivity analysis, repeating the main Cox regression but restricting to individuals 65 + years old when they received a diagnosis of MASLD. All analyses were performed using R (version 4.1.1) and the packages Survival, Survminer, and cmprsk.

### Ethical statement

The research in this study was performed in accordance with both the Declaration of Helsinki and the regulations of the Danish Data Protection Agency. Ethical approval is not required for register-based studies in accordance with Danish legislation, and no agreement of informed consent is required.

Human Ethics and Consent to Participate declarations: not applicable.

## Results

### Population characteristics

**Fig. 1:**
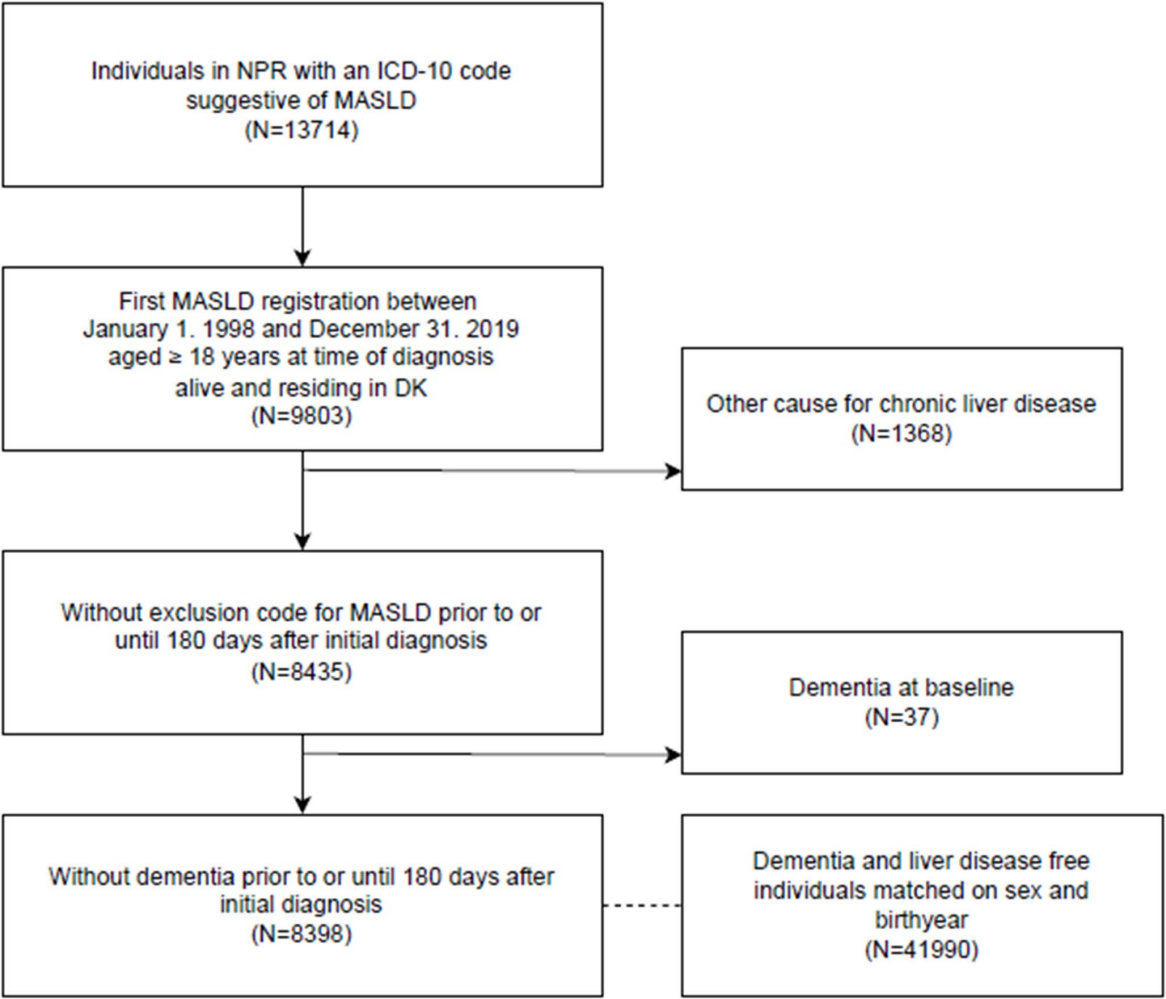
Flowchart of study population selection

A total of 8,398 individuals with MASLD were included in the study, with 41,990 references without liver disease, matched on birth year and sex (see Fig. 1). Age group, sex and distributions are displayed in Table 1 along with other baseline covariates. In brief, individuals with MASLD were two to four times more likely to have type 2 diabetes, hypertension, and dyslipidemia. There was also a doubling in the frequency of depression and heart disease, but not of stroke. There was no difference in frequency of cohabitation, but individuals with MASLD were more likely to be in lower income brackets and have lower educational attainment. Individuals with MASLD were also more likely to have a high frequency of health care contacts. A total of 174 individuals with MASLD and a total of 641 references developed dementia during follow-up, with most events (745 out of 815) happening above age 65.

**Table 1:** Baseline characteristics of MASLD and references

	MASLD	References
Number (risk time years)	8398 (91096)	41,990 (457660)
Male sex, n (%)	3865 (46)	19,325 (46)
Age group, n (%)		
< 50	3728 (44)	18,620 (44)
50–59	2223 (26)	11,119 (26)
60–69	1717 (20)	8554 (20)
70–79	630 (8)	3202 (8)
> 79	100 (1)	495 (1)
Type 2 diabetes, n (%)	1313 (16)	1730 (4)
Hypertension, n (%)	3677 (44)	10,182 (24)
Dyslipidemia, n (%)	2044 (24)	4960 (12)
Heart disease, n (%)	1182 (14)	3234 (8)
Stroke, n (%)	232 (3)	754 (2)
Depression, n (%)	629 (7)	1230 (3)
Cohabitation, n (%)		
Living alone	2647 (32)	12,974 (31)
Living with a partner	5748 (68)	28,928 (69)
NA	3 (0)	88 (0)
Education, n (%)		
Primary	3055 (36)	12,108 (29)
Secondary	3921 (47)	19,373 (46)
Tertiary	1422 (17)	10,509 (25)
Income, n (%)		
Quartile 1	2599 (31)	10,039 (24)
Quartile 2	2207 (26)	9854 (23)
Quartile 3	1929 (23)	10,518 (25)
Quartile 4	1592 (29)	10,854 (26)
NA	71 (1)	725 (2)
Health care utilization, n (%)		
Low	1118 (13)	17,043 (41)
Medium	2503 (30)	14,175 (34)
High	4777 (57)	10,772 (26)
Chronic Obstructive Pulmonary Disease, n (%)	290 (3)	731 (2)

### All-cause dementia risk

**Fig. 2:**
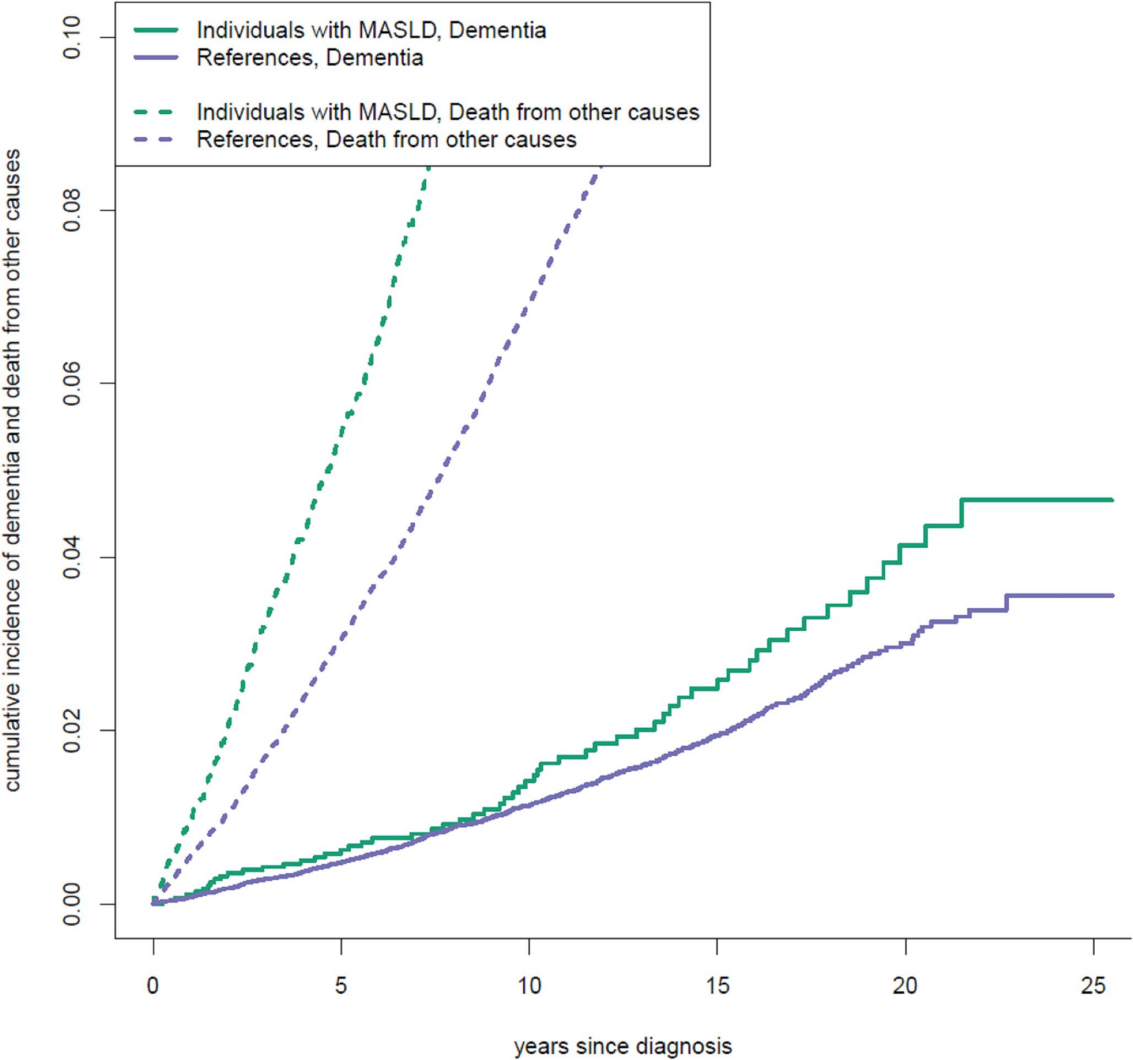
Cumulative incidence plots for all-cause dementia, and competing events (death from other causes) in individuals with metabolic dysfunction-associated steatotic liver disease (MASLD) and in references

The cumulative incidences of dementia and death from other causes are displayed in Fig. 2. Individuals with MASLD had an increased cumulative incidence of dementia, which only became apparent 8 years after diagnosis. At all times, the cumulative incidence of competing events was higher for individuals with MASLD than for matched references. Crude incidence rates for dementia were higher in individuals with MASLD than in the reference population, with comparable relative rates for both male and female patients. For individuals aged 65 and above, the incidence rate for dementia was 59.0 per 10,000 person-years in individuals with MASLD and 41.9 in matched references (Table 2).

**Table 2:** Incidence rates and hazard ratios with 95% CI from stratified Cox regression on dementia in individuals with metabolic dysfunction-associated steatotic liver disease (MASLD) and references

	IR per 10,000 person-years (95% CI)		Model 1	Model 2	Model 3
	MASLD	References	HR (95% CI)	HR (95% CI)	HR (95% CI)
*From MASLD*^*a*^					
Total population	19.1 (16.4–22.2)	14 (13.0–15.1)	1.40 (1.17–1.67)	1.22 (1.01–1.47)	1.12 (0.92–1.36)
Male	13.2 (10.0–17.1)	10.3 (9.0–11.7)	1.32 (0.96–1.81)	1.27 (0.91–1.77)	1.24 (0.87–1.77)
Female	24.4 (20.2–29.2)	17.3 (15.7–19.0)	1.43 (1.15–1.78)	1.22 (0.97–1.54)	1.12 (0.88–1.42)
*From MASLD or age 65*^*b*^					
Total population	59.0 (50.2–73.8)	41.2 (38.0–44.7)	1.42 (1.18–1.71)	1.26 (1.03–1.53)	1.16 (0.94–1.42)
Male	56.3 (18.5–27.2)	39.9 (34.4–46.0)	1.42 (1.01–1.98)	1.37 (0.95–1.96)	1.41 (0.97–2.05)
Female	60.3 (49.5–72.9)	41.9 (37.9–46.3)	1.42 (1.13–1.78)	1.24 (0.97–1.57)	1.12 (0.87–1.44)

**Model 1**: Crude model. **Model 2**: Adjusted for diabetes, hypertension, dyslipidemia, heart disease, and depression. **Model 3**: Further adjusted for cohabitation, income, education, and health care utilization. **a**: Counting risk time from MASLD index date. **b**: Counting risk time from age 65 or MASLD index date (whichever came later) in individuals free from dementia at the specified date. **IR** = incidence rate (per 10 000 person-years)

In the unadjusted Cox model, MASLD was associated with a significantly increased HR for dementia of 1.40 (95% CI: 1.17–1.67). This effect attenuated when adjusting for comorbidities. When further adjusting for socioeconomic confounders, the association was attenuated to a HR of 1.12 (95% CI: 0.92–1.36), which was not statistically significant. Restricting follow-up to time at risk from age 65 and above increased the effect size minimally to HR of 1.16 (95% CI: 0.94–1.42), which was also nonsignificant. Selecting for individuals diagnosed with MASLD only after the age of 65 lowered estimates slightly (supplementary). When stratifying on sex, the HR for male individuals with MASLD compared to male references was less affected by adjustment (HR 1.32 (95% CI: 0.96–1.81) in crude and HR 1.24 (95% CI: 0.87–1.77) in adjusted models), and generally higher than that of female individuals with MASLD compared to female references (HR 1.12 (95% CI: 0.88–1.42) in adjusted model) (Table 2).

**Fig. 3:**
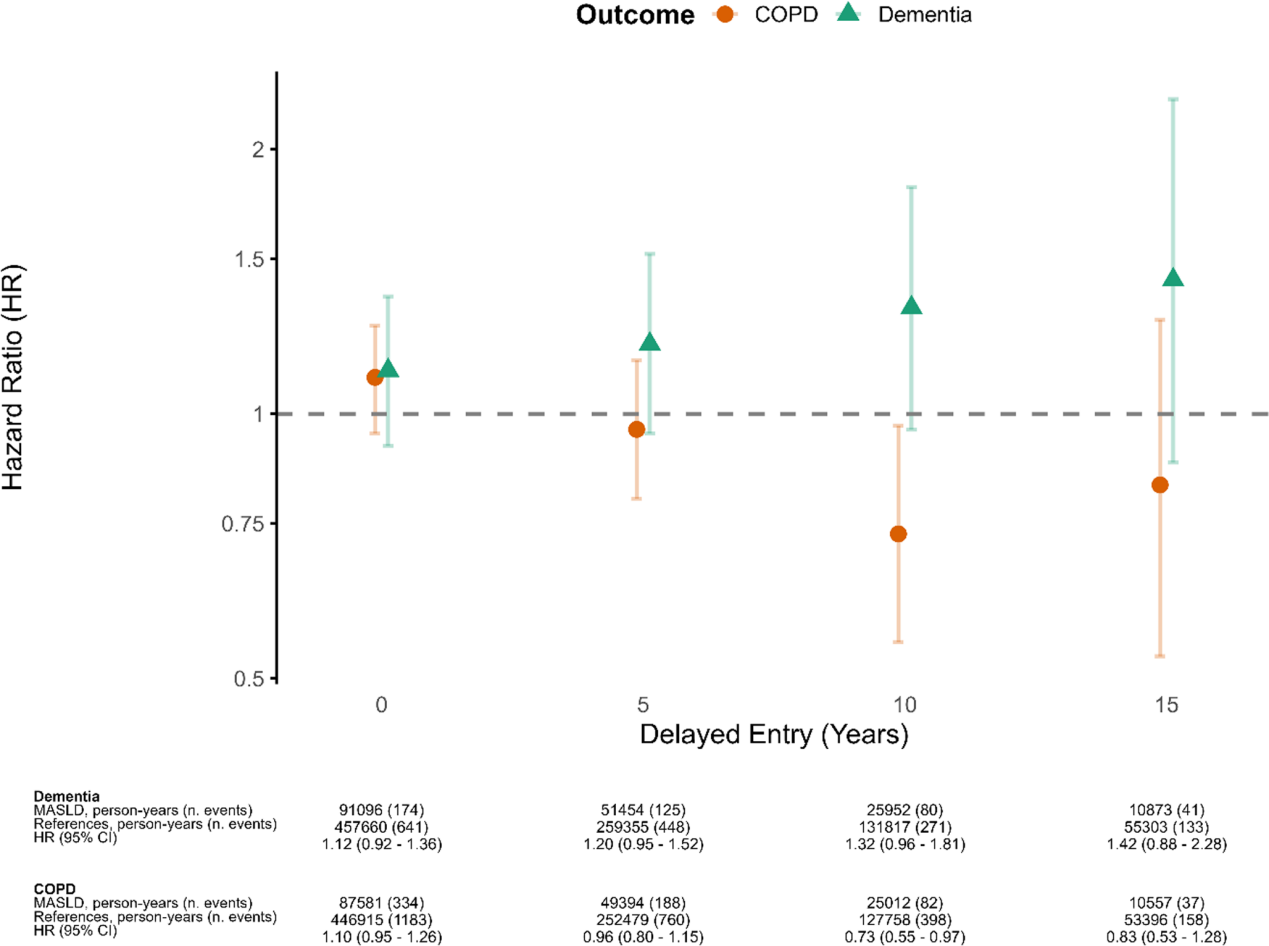
Fully adjusted stratified Cox models with varying degrees of delayed entry and dementia as outcome. Delayed entry corresponds to conditioning on censor and event-free survival until a specified time point and then setting the start time to that time point. Models are adjusted for diabetes, hypertension, dyslipidemia, heart disease, depression, cohabitation, income, education, and health care utilization

Delayed entry Cox regression, with stepwise increments of five years, and excluding individuals from analysis who either developed dementia or were censored before this time point, showed widened CIs with each increase, rendering all results nonsignificant. For every 5-year increase in the entry delay, the HR for individuals with MASLD consistently increased to 1.42(0.88–2.28) at 15 years delayed entry (Fig. 3).

### COPD and all-cause mortality

Due to COPD diagnosis before index date, we excluded 290 individuals with MASLD and 731 references. When replacing dementia with all-cause mortality, the HR for individuals with MASLD was 1.56 (95% CI: 1.45–1.68), which was statistically significant. When instead replacing dementia with COPD, the HR for individuals with MASLD was 1.10 (95% CI: 0.95–1.26), which was both comparable to that for dementia and non-significant. When introducing delayed entry, the HR for COPD in individuals with MASLD lowered, which was opposite to that of dementia (Fig. 3).

### Vascular dementia

Because the mechanisms by which MASLD could affect dementia risk are most likely vascular, we performed a sensitivity analysis only looking at vascular dementia as the outcome. A total of 28 individuals with MASLD and 105 matched references developed vascular dementia. Due to the small number of total events, we did not perform any sex stratified analyses. Cumulative incidences of vascular dementia are displayed in Supplementary 4. Cumulative incidence of competing events is not displayed due to the difference in magnitude, but is largely comparable to that of dementia. There was no apparent difference in the cumulative incidence of vascular dementia specifically but an increased cumulative incidence of competing events. Crude incidence rates were slightly higher in individuals with MASLD compared to the reference population. In the regression model HR attenuated from 1.22 in both crude models (95% CI: 0.78–1.92 in main model, 0.70–2.14 in model for individuals over 65 years) to 0.92 (95% CI: 0.48–1.78) in the fully adjusted model for individuals over 65 years, and all models were non-significant (supplementary 5).

## Discussion

In this large, population-wide matched cohort study of real-world health data, we observed an increased risk of dementia in individuals diagnosed with MASLD compared to matched individuals, but the association largely disappeared when adjusting for comorbidities and socioeconomic factors.

Our findings concur with prospective studies (Xiao et al. 2022; Huang et al. 2023; Wang et al. 2022), but not with previous registry-based studies (Jeong et al. 2022; Shang et al. 2022)A recent Swedish study with similar comorbidity adjustment reported incident MASLD to be associated with dementia, and found results to be stable when conditioning on 5 and 10 years of follow-up (Shang et al. 2022). In our analysis, additional adjustment for socioeconomic status attenuated this association, and delayed entry analyses indicate potential reverse causation, reinforcing the need for caution when interpreting observational associations between MASLD and dementia. Dementia is often preceded by a long prodromal phase of cognitive decline, which commonly includes disease-related weight loss. Prior research has demonstrated that this weight loss can begin several years before a formal dementia diagnosis (Singh-Manoux et al. 2018). Since MASLD is strongly influenced by body weight (Koutoukidis et al. 2021), this raises the possibility of reverse causation, where preclinical dementia-related weight loss could reduce the likelihood of recieving a MASLD diagnosis, either by leading to resolution of existing but undiagnosed MASLD or by lowering the risk of developing MASLD altogether. If the true causal association between MASLD and dementia risk is positive, this process would be expected to bias observed estimates in a negative direction, attenuating the association and potentially masking a real effect. Consequently, a null or weak association in this setting should not necessarily be interpreted as evidence against a causal relationship.

Furthermore, previous prospective studies have found that while MASLD may not always be associated with increased risk of diagnosed dementia, it may still contribute to accelerated cognitive decline (Wang et al. 2022; Cushman et al. 2023). In our study, the association between MASLD and dementia became stronger when we conditioned on dementia-free survival time after MASLD diagnosis, supporting the possibility that preclinical dementia may obscure the true relationship between MASLD and later cognitive outcomes. This finding differs from the aforementioned Swedish study, but may in part be explained by the fact that we adjusted for health care utilization (number of recent health care contacts) at baseline, which may have removed some detection bias that could counteract the effect of reverse causation (Shang et al. 2022).

Previous registry-based studies have largely only included individuals diagnosed with MASLD at age 65 and above, due to the lower reliability of dementia cases below this age. However, most individuals who get a MASLD diagnosis are diagnosed before this age and can also develop dementia after turning 65. Restricting to late-life MASLD by eliminating these individuals may introduce selection bias. Our findings showed that including risk time before age 65 does not meaningfully impact relative risk measurements, compared to including all risk time. This is likely because while diagnoses of dementia in this age period may be imprecise, they are also rare. Restricting the population with MASLD to those diagnosed after age 65 greatly limited the observed number of individuals and slightly attenuated the results, which may be an indication of some small selection bias in only examining those diagnosed late in life.

At the same time, individuals with MASLD only had a small and not statistically significantly increased risk of being diagnosed with COPD. As there is no known mechanism by which MASLD should be able to cause COPD, and the MASLD to COPD associations share confounders with the MASLD to dementia association, such as smoking and alcohol consumption, any strong association would show that results are likely to be biased. Unlike the association with dementia, the relationship between MASLD and COPD attenuated and reversed direction after conditioning on COPD-free survival. Given the established links between smoking, alcohol consumption, and dementia, this may suggest that any residual confounding from these factors likely biases the estimated MASLD-dementia association downward rather than upward (Xu et al. 2017; Reitz et al. 2007).

We also specifically investigated vascular dementia, as MASLD is associated with poor vascular health. Results were similar to those of all-type dementia, but were impacted by a low number of events. It is likely that vascular dementia is more underreported than dementia in general, as treatment options for this subtype are more limited.

Recent observational evidence suggests that glucagon-like peptide-1 (GLP-1) receptor agonists, which are currently being investigated for their treatment potential in MASLD (Sanyal et al. 2025), may also offer protective effects against dementia. Proposed mechanisms include reductions in neuroinflammation, oxidative stress, and amyloid-beta accumulation (Wang et al. 2024; Wium-Andersen et al. 2019; Nørgaard et al. 2022). In fact, ongoing clinical trials are now assessing GLP-1 receptor agonists in patients with Alzheimer’s disease (Cummings et al. 2025), which may clarify their potential for neuroprotection (Newsome et al. 2024).

The strengths of this study include its large sample size, high level of data completeness, and extended follow-up time. The use of Registry data minimizes loss to follow-up and ensures accurate assessment of outcomes, and eliminates participation bias. We were also able to adjust for a wide set of confounding variables, including both comorbidities and socioeconomic factors that may influence the association between MASLD and dementia. The use of various sensitivity analyses also allowed us to assess the changes in effect sizes when separating the exposure and outcome time-wise, to address possible effects from reverse causation, and to assess possible residual confounding through the use of a negative control outcome.

This study also has several limitations. As MASLD is often asymptomatic, it is also underdiagnosed; misclassification of MASLD in the form of underdiagnosing will lead to some bias towards the null (Lindvig et al. 2022; Castera et al. 2025). This underdiagnosis may have been particularly pronounced among the earliest included individuals, when the relevant ICD-10 codes were used less consistently, thereby introducing some calendar-time-dependent misclassification. Because our delayed-entry design places relatively greater weight on individuals with longer follow-up, who are also those included earliest, any such misclassification would be expected to bias our estimates further toward the null. Consequently, the true effect sizes may be larger than those observed. It should be noted that the registry-based approach is likely to reflect the actual cohort of MASLD patients in the health care system, and that such misclassification is likely to extend into clinical practice. It was also not possible to adjust for obesity or smoking at baseline, which may be contributing factors in dementia development. However, negative control analysis suggests that the impact of smoking in this context is minimal and may in fact make associations smaller.

In conclusion, this registry-based study did not observe an increased risk of incident dementia in those diagnosed with MASLD when adjusting for confounding variables, including age, sex, metabolic comorbidities, and socioeconomic factors. Thus, this study does not support increased risk assessment in individuals with MASLD compared to individuals with comparable metabolic and socioeconomic status. However, timing between MASLD and Dementia may reveal a more complex association, and MASLD may be associated with an increased long-term risk.

## Supplementary Information

Below is the link to the electronic supplementary material.

Supplementary File 1 (PDF 215 KB)

## Data Availability

Data can be made available upon reasonable request. Access can be granted through collaborative agreements and directly via Statistics Denmark and the Danish Health Data Authorities. Due to Danish data protection regulations, individual-level registry data cannot be shared or deposited in public repositories. Instead, approved researchers can access the data through secure servers hosted by Statistics Denmark and the Danish Health Data Authorities after obtaining the necessary permissions. Please contact Professor Majken K Jensen (maje@sund.ku.dk) for further information.

## Abbreviations

MASLD: Metabolic Dysfunction-Associated Steatotic Liver Disease
COPD: Chronic Obstructive Pulmonary Disease
HR: Hazard Ratio
DNPR: Danish National Patient Registry
PCRR: Psychiatric Central Research Registry
ICD: International Classification of Diseases
ATC: Anatomical Therapeutic Chemical classification
CI: Confidence Interval
GLP-1: Glucagon-Like Peptide-1
