# Supplementary material for: Long-term dementia risk in metabolic dysfunction-associated steatotic liver disease: a population-based study
Source: Metab Brain Dis. 2026 Feb 2;41(1):25. doi: 10.1007/s11011-026-01796-x (PMC12864253; doi:10.1007/s11011-026-01796-x)
Supplement: Supplementary file 1 — (PDF 215 KB) [file 11011_2026_1796_MOESM1_ESM.pdf]

Supplementary 1. Codes used for exclusions

	Groups excluded	ICD-10	ICD-8	SNOMED-CT (in liver biopsy)
Viral Hepatitis	Hepatitis B, Hepatitis C	B(16-19)	700(0-9)	S0520(0-2), S05204, S05252, S05255
Alcohol	Alcoholic liver disease, Other alcohol related diseases, and indicators of excessive alcohol consumption	E224, G312, G721, I426, K292, K70, K852, K860, T500A, X65, Z502, Z714, Z721	291, 303, 57109, 57110, 57710, 979	
Other substance abuse	Abuse of substances other than alcohol, excluding tobacco	F(10-19)	304(0-9)9	
Other Causes for Chronic Liver Disease	Hemochromatosis, Wilson's disease, Alfa-1-antitrypsin deficiency, glycogen storage diseases, Budd-Chiari, Toxic liver disease, Autoimmune hepatitis, primary/secondary biliary cirrhosis, Congestive hepatopathy, HIV/AIDS, cholangitis, celiac disease, hepatic cancers.	B20, B(22-24), E740, E831A, E880B, E830B, I820, K71, K732, K744, K745, K75(1-4), K761, K830, K900	27109, 27119, 57191(0-2), 27329, 155(0-1)9, 15589, 19789, 57504, 26900, 27080, 27339, 45301, 5730(1-2), 7983	M49580, M49590, M49592, M49691, S10700, S11920, S63530, S63580, S6360(5-6), S63608, S63610, S63630, S97250, SYY610, SYY636<br><br>And any Code beginning with M8 or M9 and ending in 3, 6, or 9

Supplementary 2. Codes used to define dementia

	ICD-8	ICD-10	ATC
Dementia, all types	290*, 29309, 29319	F00, G30, F01, F02, F039, G318, G319	N06DX01, N06DA02, N06DA03, N06DA04
Vascular dementia		F01	

Supplementary 3. Codes used for comorbidities

	ICD8	ICD10	ATC
Diabetes			A10
Hypertension			C02A, C02DB, C02DD, C03, C07, C08, C09A, C09C, G04CA
Dyslipidemia			C10
Stroke	431, 432	I61, I63, I64	
Depresson	29(5-9)	F3, F412	
Heart disease	400, 425, 427, 428, 41(0-3)	I110, I2(0-4), I42, I43, I48, I50, I517	
copd	491, 492	DJ440, DJ441, DJ448, DJ449	

Supplementary 4. Cumulative incidence plot for vascular dementia in individuals with Metabolic dysfunction-associated steatotic liver disease (MASLD) and matched references

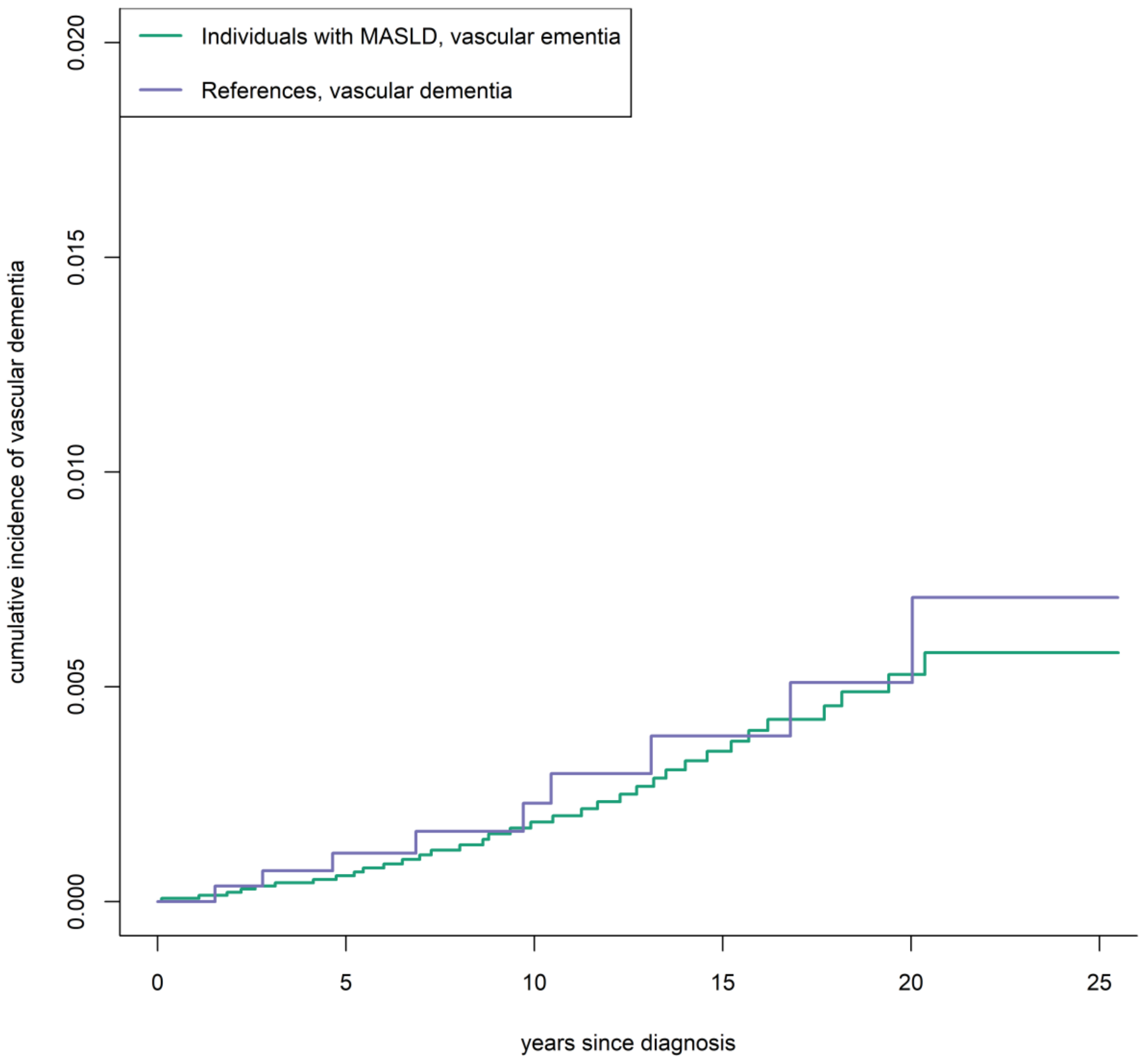

Supplementary 5. Incidence rates and Hazard ratios with 95% CI from stratified Cox regression on vascular dementia in individuals with Metabolic dysfunction-associated steatotic liver disease (MASLD) and references.

	IR per 10000 person-year (95% CI)		Model 1	Model 2	Model 3
	MASLD	References	HR (95% CI)	HR (95% CI)	HR (95% CI)
From MASLD[a]	3.1 (2.0 – 4.4)	2.3 (1.9 – 2.8)	1.22 (0.78 – 1.92)	1.15 (0.7 – 1.87)	1.07 (0.64 – 1.79)
From MASLD or age 65[b]	9.0 (5.8 – 13.3)	7.0 (5.7 – 8.6)	1.22 (0.7 – 2.14)	1.12 (0.61 – 2.07)	0.92 (0.48 – 1.78)

Model 1: Crude model. **Model 2:** Adjusted for diabetes, hypertension, dyslipidemia, heart disease, and depression. **Model 3:** Further adjusted for cohabitation, income, education, and health care utilization. **a**: Counting risk time from MASLD index date. **b**: Counting risk time from age 65 or MASLD index date (whichever came later) in individuals free from dementia at the specified date

Supplementary 6. Hazard ratios for MASLD and all-cause dementia, restricting to individuals diagnosed at age 65 and above and their matched references

Model 1	Model 2	Model 3
HR (95% CI)	HR (95% CI)	HR (95% CI)
1.23 (0.96 – 1.56)	1.08 (0.84 – 1.39)	1.00 (0.78 – 1.30)

Model 1: Crude model. **Model 2:** Adjusted for diabetes, hypertension, dyslipidemia, heart disease, and depression. **Model 3:** Further adjusted for cohabitation, income, education, and health care utilization.
